# Lipoplatin Treatment in Lung and Breast Cancer

**DOI:** 10.1155/2011/125192

**Published:** 2010-12-21

**Authors:** Manuela Fantini, Lorenzo Gianni, Carlotta Santelmo, Fabrizio Drudi, Cinzia Castellani, Alessandra Affatato, Mario Nicolini, Alberto Ravaioli

**Affiliations:** ^1^Department of Oncology and Oncohematology, Infermi Hospital, 2 Settembrini Street, 47900 Rimini, Italy; ^2^Department of Oncology, Cervesi Hospital, 1 Beethoven Street, 47841 Cattolica, Italy; ^3^The Cancer Institute of Romagna IRST, 40 Piero Maroncelli Street, 47041 Meldola, Italy

## Abstract

The introduction of cisplatin in cancer treatment represents an important achievement in the oncologic field. Many types of cancers are now treated with this drug, and in testicular cancer patients major results are reached. Since 1965, other compounds were disovered and among them carboplatin and oxaliplatin are the main Cisplatin analogues showing similar clinical efficacy with a safer toxicity profile. Lipoplatin is a new liposomal cisplatin formulation which seems to have these characteristics. Lipoplatin was shown to be effective in NSCLC both in phase 2 and phase 3 trials, with the same response rate of Cisplatin, a comparable overall survival but less toxicity. A new protocol aiming to elucidate the double capacity of Lipoplatin to act as a chemotherapeutic and angiogenetic agent in triple-negative breast cancer patients is upcoming.

## 1. Introduction

In the present scenario of cancer medical treatment, Platinum compounds and Cisplatin are considered important drugs by clinicians and are useful in many types of neoplastic diseases. 

Their use is especially devoted to the treatment of epithelial malignancies, but also lymphoma and sarcoma could be sensitive to these drugs. From its discovery in 1965, cisplatin had a milestone achievement in clinical oncology, and has saved thousands of lives of testicular cancer patients.

As a matter of fact, Cisplatin is recommended for the treatment of metastatic testicular, ovarian, and transitional bladder cancer and also nonsmall cell lung cancer (NSCLC), cervical cancer, head and neck cancer, esophageal cancer, gastric cancer, breast cancer and many other malignancies.

The principal limitations for its use are due to its possible severe toxicities, especially nephrotoxicity, neuropathy, ototoxicity, and hematological toxicities. The most important significant toxicity is linked to renal damage. For this reason, many other compounds have been searched in order to overcome this problem. In the recent years, two other Cisplatin-like drugs were introduced in clinical practice, such as carboplatin and oxaliplatin. Carboplatin is mainly used in the treatment of NSCLC and ovarian cancer while Oxaliplatin is mainly used in colorectal carcinoma.

These two drugs are burdened by different toxicities, such as hematological toxicity for Carboplatin and neurotoxicity for Oxaliplatin.

Moreover, their antitumor efficacy is very different from Cisplatin, whose use cannot be substituted in some pathologies like testicular cancer and lung cancer, but also bladder and biliary tumors.

For these reasons, the research of different compounds having platinum efficacy without the limitation of its general toxicity are currently underway.

Lipoplatin was recently introduced in clinical practice after the observation of its efficacy and mild toxicity.

## 2. The Lipoplatin Drug

Liposomes are an important advanced technology that allows to deliver active molecules to the disease' site one and nowadays several formulation are entered in clinical practice. 

Liposome tecnology has reached the “second generation” with long circulating liposome built, modulating lipid composition size and charge of the vescicles [1]. 

Lipoplatin TM is a new liposoma cisplatin formulation developed in order to reduce the systemic toxicity of cisplatin, enhancing tumor targeting [2]. 

This molecule measures 110 nm and is composed of a lipid shell made of dipalmitoyl-phosphatidyl glycerol (DPPG), soy, phosphatidyl-choline (SPC/3), cholesterol (CHOL), methoxy-polyethylene, and glycol-distearoyl phosphatidylethanolamine lipid conjugate (mMPEG 2000-DSPE), containing a central core of Cisplatinum with a ratio of Cisplatinum to lipids of 8.9% Cisplatin and 91.1% total lipids. A PEG polymer coating is added to the molecule.

This modified formulation is not detected by macrophages and immune cells, and, therefore, it circulates longer in body fluids and tissues, with accumulation preferentially in primary tumor sites and metastases. 

Preclinical trials have demonstrated the ability of this molecule to be concentrated up to 50 times more in malignant tissues than in normal tissues [2].

While Cisplatin efflux is regulated by at least two copper of efflux transporter, Lipoplatin is proposed to bypass this process. Thus Lipoplatin might have application even in previously Cisplatin-pretreated patients who have developed resistant tumours.

## 3. Phase I Studies and Lipoplatin Pharmacokinetics

In a Phase I study on 27 patients with pretreated (2nd- or 3rd- line treatment) stage IV cancer (19 pancreatic carcinoma, 6 renal cell carcinoma, 1 gastric cancer, and 1 squamous cell carcinoma of the head and neck SCCHN), Lipoplatin dose was escalated from 25 to 125 mg/m^2^, and it was administered as an 8-hour infusion diluted in 1 L 5% dextrose, repeated every 2 weeks without need for pre or posthydration. Three patients were also treated at higher-dose levels, one at 200, 250, and 300 mg/m^2^, respectively [2]. There were no deaths or other serious adverse events (SAEs). Lipoplatin had mild hematological and gastrointestinal toxicity, and it did not show any nephrotoxicity, neurotoxicity or ototoxicity, nor caused hair loss. Grade 1 and 2 myelotoxicity (neutropenia) and grade 1 and 2 GI tract toxicity (vomiting) were observed only at the dose of 125 mg/m^2^. The maximum tolerated dose (MTD) was not reached even when the dose was increased up to 350 mg/m^2^ in one patient as a single infusion. No other toxicity was observed even with repeated doses. At the beginning of the infusion, 8 (29.6%) of 27 patients described acute severe epigastric and back pain that lasted for about 5 minutes and subsided spontaneously without analgesic administration. This pain is characteristic of other liposomal drugs as well. 

Although measurement of the response rate was not a primary goal of the study, 3 (11.1%) of 27 patients were recorded to have achieved a partial response; of the remaining 24 patients, 14 (51.9%) achieved stable disease and clinical benefit in a followup of 2–5 months.

As for Pharmacokinetics, measurement of platinum levels in the plasma of patients as a function of time showed that maximum platinum level in the plasma (Cmax) was attained at 6–8 hours after the start of the infusion. Half-life (*T*_1/2_) was 37–50 hours depending on the dose, in comparison with the 6 hours for cisplatin. Excretion of platinum in the urine reached a maximum during the infusion period and declined thereafter. Following a 100 mg/m^2^ infusion, 9.1% of the dose was excreted in the urine during the 8-hour infusion, and 16.8% and 10% of the dose were excreted in the urine during the following 16 and 24 hours, respectively. During the third day, an additional 4.8% of the dose was excreted in urine. Overall, 40.7% of total platinum was excreted in urine during the 3 days after the start of the infusion. Urine excretion slowed after the third day. 

Another open-label, dose-escalation, phase I clinical study was conduced to identify the MTD and investigate the pharmacokinetics of Lipoplatin when combined with gemcitabine, as 2nd-or 3rd- line treatment in patients with advanced NSCLC [3].

Thirteen patients with stage IIIb (*n* = 2) or stage IV (*n* = 11) NSCLC were enrolled in the study. Lipoplatin was given as a 4-hour IV infusion on days 1 and 8 of a 21-day cycle. Gemcitabine 1000 mg/m^2^ was administered as a 30-minute IV infusion prior to the Lipoplatin infusion on the same days. Doses were 100 mg/m^2^ (*n* = 3), 110 mg/m^2^ (*n* = 3), 120 mg/m^2^ (*n* = 3), and 130 mg/m^2^ (*n* = 4). Treatment continued until completion of 3 cycles or disease progression. Blood samples were collected at predefined time intervals after start of the infusion, and analyzed for total platinum levels. 

There were no deaths or SAEs. 75% of the patients at the 130 mg/m^2^ dose had dose reduction to 120 mg/m^2^ due to grade 2-3 myelotoxicity; no hemopoietic factors were given. Mild elevation in serum creatinine levels was observed in a single patient in the 130 mg/m^2^ dose; two more patients in the same dose with prior impaired renal function received treatment with no aggravation of their renal insufficiency. Mild gastrointestinal toxicity was also seen in the higher doses. MTD of Lipoplatin in the Lipoplatin plus gemcitabine combination was defined as the 120 mg/m^2^ dose, DLT being myelotoxicity. 

A disease-control rate of 3 (23%) of 13 was found; the median overall survival was 29 weeks (range 4–52), and the median time to progression was 12 weeks. 

An open-label, dose-escalation, phase I/II clinical study was designed to identify the MTD of Lipoplatin when combined with gemcitabine in patients with advanced pancreatic cancer refractory to previous gemcitabine-based chemotherapy [4].Twenty-four patients with stage IIIb (*n* = 5) or stage IV (*n* = 19) pancreatic adenocarcinoma were enrolled in the study. Lipoplatin was given as an 8-hour IV infusion on days 1 and 14 of a 28-day cycle. Gemcitabine 1000 mg/m^2^ was administered prior to the Lipoplatin infusion on the same days. Doses were 25 mg/m^2^ (*n* = 4), 50 mg/m^2^ (*n* = 4), 75 mg/m^2^ (*n* = 4), 100 mg/m^2^ (*n* = 4), and 125 mg/m^2^ (*n* = 4). An additional 4 patients were enrolled and treated with 100 mg/m^2^, this was defined as the MTD of this combination and the treatment schedule in gemcitabine-pretreated patients. Treatment continued until completion of 3 cycles or disease progression. 

There were no deaths or SAEs. No adverse events were observed at doses up to 100 mg/m^2^. At the 125 mg/m^2^ dose, grade 3 and 4 neutropenia was observed in 2 patients, with no associated symptoms of infection. Temporary abdominal pain that lasted for 2–4 minutes, and resolved spontaneously, was observed in 42% of the patients at the beginning of Lipoplatin infusion. No nephrotoxicity was observed at any dose. There were no other grade 2, 3, or 4 adverse events. There was no dose reduction in any of the administered drugs. MTD of Lipoplatin in the Lipoplatin plus gemcitabine combination in gemcitabine-pretreated patients was defined as the 100 mg/m^2^ dose, DLT being myelotoxicity. Preliminary objective response rate data showed a PR in 2 (8.3%) and disease stability in 14 patients (58.3%).

## 4. Phase II Studies

A number of phase II clinical studies have shown a low toxicity profile and a therapeutic efficacy of Lipoplatin against pancreatic cancer and NSCLC when combined with gemcitabine, paclitaxel, or vinorelbine (Table 1).

In one phase I-II study, 38 patients with advanced cancers (19 pancreatic, 6 NSCLC, 4 mesothelioma, 4 urinary bladder, 2 SCCHN, 2 renal, 1 prostate) were treated with Lipoplatin at 100 mg/m^2^ on days 1, 14 in combination with gemcitabine 1000 mg/m^2^ on days 1, and 8 in a 28-day cycle as 2nd or 3rd line chemotherapy. The side effects observed were mainly those expected from gemcitabine monotherapy, including myelotoxicity (thrombocytopenia, neutropenia, anaemia).

An average of 4 infusions with Lipoplatin and gemcitabine were administered; two patients had 12 infusions. There were 3 complete responses in lung lesions; in a patient with a metastatic lesion from a primary pancreatic cancer, in a patient with a metastatic lesion from a primary prostate adenocarcinoma, and in a patient with a primary stage IIIa NSCLC lesion [4]. 

Two phase II trials evaluated Lipoplatin in patients with advanced NSCLC patients who previously underwent cisplatin-based chemotherapy; in our previous experience, 19 pretreated patients (stage IV) NSCLC were treated with Lipoplatin 100 mg/m^2^ on day 1 in a 14-day cycle, as 2nd line treatment. On disease evaluation after 6 cycles, 1 patient (5.2%) had partial response, and 3 patients (15.9%) had disease stabilization. The only adverse event was grade 1 gastrointestinal toxicity (nausea/vomiting) [5]. In the second clinical report, patients with NSCLC refractory to Cisplatin were treated with Lipoplatin 120 mg/m^2^ days 1 and 8 plus gemcitabine 1000 mg/m^2^ days 1 and 8 every 3 weeks. Twenty-seven (77.8%) patients (21 males) were assessable for response and toxicity according to the WHO criteria of a median age of 70 years (41–78). Twenty-two (81.5%) patients were at stage IV at diagnosis; 14 (51.8%) patients had adenocarcinoma and 13 (48.2%) had squamous-cell carcinoma in histological type. PR was observed in 6 (22.2%), SD in 5 (18.5%), and progressive disease in 16 (59.2%) patients. With respect to hematological toxicity, grade 3-4 neutropenia was observed in six (22.2%) patients, grade 3 thrombocytopenia in one (3.7%), patient and grade 3 anemia in one (3.7%) patient. Other toxicities included grade 3-4 nausea/emesis in nine (33.3%) patients, grade 3 fever in nine (33.3%) patients, and grade 3 nephrotoxicity in one (3.7%) patient. Further toxicities such as rush, constipation, and peripheral neuropathy were rare and/or mild. Median overall time to tumor progression was 14 weeks (3–50) [6].

A phase II randomized, open-label, multicenter, safety and efficacy clinical trial compared 1st-line treatment with Lipoplatin plus gemcitabine with that of cisplatin plus gemcitabine in patients with advanced NSCLC. 88 patients with inoperable stage I-IIIa (*n* = 7), stage IIIb (*n* = 21), or IV (*n* = 60) NSCLC were enrolled in the study and assigned to the Lipoplatin (*n* = 47) or cisplatin (*n* = 41) arm. Lipoplatin 120 mg/m^2^ was given as a 6-hour IV infusion on days 1, 8, and 15 of a 21-day cycle. Cisplatin 100 mg/m^2^ was given as a 2-hour IV infusion on day 1 of a 21-day cycle. Patients in both arms received gemcitabine 1000 mg/m^2^ as a 30-minute IV infusion prior to the Lipoplatin/cisplatin infusion, on days 1 and 8 of the cycle. Treatment was continued until completion of 6 cycles, unacceptable toxicity, or disease progression. 

There were no deaths or SAEs. Three patients in the Lipoplatin group had a hypersensitivity reaction during the first Lipoplatin infusion. The commonest toxicities observed were grade I-II myelotoxicity, with similar rates between the two arms of treatment. Nevertheless, grade III-IV myelotoxicity was much more common (1.5 to 2 fold) in the cisplatin arm. Grade II-III nephrotoxicity was observed in 10.6% of Lipoplatin patients (versus 22%). Grade I nephrotoxicity was observed in approximately 40–45% of patients in either group, mostly in single infusions. Gastrointestinal toxicity was observed to be more frequent and more severe in cisplatin patients (grade II-III 61% versus 15%). Concerning other adverse events, severe asthenia and anorexia was 5 to 6 fold more common in the cisplatin-treated patients. Objective response was higher in the Lipoplatin arm (31.7% versus 25.6%), particularly amongst patients with adenocarcinoma [7]. 

Experience with Lipoplatin in phase II trials in metastatic breast cancer will be presented below [8]. Finally, Lipoplatin and concomitant radiotherapy were investigated in a Phase II study in locally advanced gastric adenocarcinomas. Patients with locally advanced gastric cancer or gastric cancer inoperable for medical reasons, or recurrent carcinomas of a performance status of 0–2 were recruited. Lipoplatin was given at a dose of 120 mg/m^2^, 5-FU at 400 mg/m^2^ (day 1), while radiotherapy was given through 3.5 Gy fractions on days 2, 3, and 4 in a 7-day schedule. Two groups of six patients received 4 and 5 consecutive cycles, respectively. Twelve of twenty planned patients in this study have completed treatment. No WHO grade 3 or 4 nephrotoxicity, anemia, asthenia, or neuropathy were noted, except of grade III neutropenia in 1 (8%) of 12 patients. A net improvement of the performance status was recorded at 2 months after the end of therapy. The response rates assessed with CT scans, endoscopy, and biopsies confirmed 33% (2/6) complete remission, 3 (50%) of 6 PR in patients treated with four cycles, and 4 (80%) of 5 complete remission in patients treated with five cycles [9].

## 5. Phase III Trials

The first phase III clinical trial started in Germany in December 2003 and is still recruiting patients. It is a randomized, open-label, multicenter safety and efficacy study in patients with advanced SCCHN [10]. It compares the safety and efficacy of treatment with Lipoplatin plus 5-Fluorouracil (5-FU) with that of cisplatin plus 5-FU. Lipoplatin 100 mg/m^2^ is given as a 6-hour IV infusion on days 1, 8, and 15 of a 21-day cycle. Cisplatin 100 mg/m² is given as a 1-hour IV infusion on day 1 of the cycle. Patients in both arms receive continuous infusion of 5-FU 1000 mg/m²/d on days 1–5 of the cycle. Treatment is continued until completion of 6 cycles, unacceptable toxicity, or disease progression. The purpose of the study is to show that treatment with Lipoplatin plus 5-FU gives comparable response and overall survival to cisplatin plus 5-FU, but is at the same time better tolerated, and patients have a better quality of life. Preliminary data show that the toxicity profile is much lower in the Lipoplatin Arm, with similar clinical benefit. 

A second Phase III clinical trial started in Greece in April 2005 and is still recruiting patients. It is a randomized, open-label, multicenter safety and efficacy study in patients with advanced NSCLC. The objective of this trial is to compare the safety and efficacy of 1st line treatment with Lipoplatin plus gemcitabine with that of cisplatin plus gemcitabine. Lipoplatin 120 mg/m^2^ is given as a 6-hour IV infusion on days 1, 8, and 15 of a 21-day cycle. Cisplatin 100 mg/m^2^ is given as a 2-hour IV infusion on day 1 of a 21-day cycle. Patients in both arms receive gemcitabine 1000 mg/m^2^ as a 30-minute IV infusion prior to the Lipoplatin/cisplatin infusion, on days 1 and 8 of the cycle. Treatment is continued until completion of 6 cycles, unacceptable toxicity, or disease progression. The purpose of the study is to show that Lipoplatin, as 1st line treatment, is not inferior to cisplatin when combined with gemcitabine (assessed by overall survival), and is better tolerated. Preliminary results were presented at the ASCO meeting in 2007 and at the ESMO meeting in 2009 and show that Lipoplatin may have a better safety and therapeutic profile than cisplatin, when combined with gemcitabine, in advanced NSCLC, especially against adenocarcinomas [11, 13]. 

Finally, the results of a phase III safety and efficacy clinical study that compared Lipoplatin plus paclitaxel with cisplatin plus paclitaxel, as 1st line treatment in NSCLC have been recently published. Lipoplatin 200 mg/m^2^ or cisplatin 75 mg/m^2^ was given on day 1 of a 14-day cycle, combined with paclitaxel 135 mg/m^2^. Treatment was continued until completion of 9 cycles, unacceptable toxicity, or disease progression [12]. Patients in Lipoplatin arm showed statistically significant lower nephrotoxicity, grade 3 and 4 leucopenia, grade 2 and 3 neuropathy, nausea, vomiting, and fatigue. There was no significant difference in median and overall survival and TTP between the two arms; median survival was 9 and 10 months in Lipoplatin and cisplatin arms, respectively, and TTP was 6.5 and 6 months, respectively. However, patients with adenocarcinoma or undifferentiated NSCLC treated with Lipoplatin had better response rate (59.5% versus 42.5%) and median survival (10 months versus 8 months) after 18 months; the number of surviving patients was doubled for the Lipoplatin arm compared with Cisplatin arm (Table 2) [14].

## 6. Lipoplatin in the Treatment of Lung Cancer

There are many experiences in the use of Lipoplatin in lung cancer, expecially NSCLC, in Phase 2 and 3 trials. From these trials, we can underline that the drug is charged by a mild toxicity, without the effects on kidney and nervous system characteristic of many other platinum compounds.

Also our group performed a phase II trial in 19 patients with NSCLC pre-treated with Cisplatin-gemcitabine (31.5%), Carboplatin-gemcitabine (31.5%), gemcitabine-docetaxel (31.5%), and gemcitabine (5.5%). The dose of Lipoplatin used was of 100 mg/m^2^ diluted in 500 ml of 5% Dextrose in 8-hour infusion every 2 weeks [5]. Our results confirm what has been previously reported in the literature and provide evidence on the safer toxicity profile of Lipoplatin and its clinical efficacy.

We observed, in these heavily pre-treated patients, one partial response and three stable disease (21.1%), MTTP of 4 months (range 1–21), median survival time of 7.2 months, and one-year survival rate of 16.6 %.Very little toxicity was observed with anemia, mucosities, nausea and vomiting, and asthenia, all not superior to grade 1-2. 

In contrast, few authors have reported more severe toxicities. Anevlavis et al. [6] treated 27 platinum-refractory patients were treated with Lipoplatin 120 mg/m^2^ days 1, 8 plus Gemcitabine 1000 mg/m^2^ every 3 weeks with 37% of PR and SD.

In this experience, the author described some grade 3-4 toxicities, but not neuropathy and only a case of grade 3 nefrotoxicity.

The two randomized trials in NSCLC with Lipoplatin as first line treatment also report interesting results. Mylonakis et al. treated 88 patients in a phase II randomized trial comparing Lipoplatin or Cisplatin (Lipoplatin 120 mg/mq as a 6-hour ev infusion 1, 8, and 15 of a 21-day cycle) [7].

Cisplatin 100 mg/m^2^ was given as a two-hour ev infusion of a 21-day cycle. In both arms, patients received gemcitabine 1000 mg/m^2^ on day 1, 8 of the cycle. Also in this experience, lipoplatin showed less myelotoxicity and grade 2-3 nephrotoxicity with less severe asthenia and anorexia. Objective response was higher in the Lipoplatin arm (31.7% versus 25.6%) particurarly among patients with adenocarcinoma.

Finally, the result of the phase III clinical study compared Lipoplatin plus paclitaxel versus Cisplatin plus Paclitaxel as first line treatment (Lipoplatin 200 mg/m^2^ or Cisplatin 75 mg/m^2^ on day 1 of a 14-day cycle combined with Paclitaxel 135 mg/m^2^). Also in this experience, Lipoplatin arm showed a lower nephrotoxicity, myelotoxicity, and neurophaty [12, 14]. No difference was observed in median overall survival or TTP but patients with adenocarcinoma or undifferentiated NSCLC treated with lipoplatin had a better response rate (49.5% versus 42.5%) and median survival (10 against 8 months). After 18 months the number of surviving patients was doubled for the lipoplatin arm compared with the Cisplatin arm.

In conclusion, we observed that Lipoplatin is a drug with the same result of Cisplatin in NSCLC with less toxicities. The observations of good responses in the histology of adenocarcinoma and undifferenciated NSCLC deserve further experiences, and a phase III trial in these particular type of cancers is worthy.

## 7. Lipoplatin in Breast Carcinoma

There are very few studies in advanced breast carcinoma. The experience coming from phase I and II trials is rare and anecdotal. The paper of Farhat is the only published experience [8].

The author treated 30 patients with metastatic breast disease without prior chemotherapy in an advanced setting with Lipoplatin 120 mg/m^2^ in days 1, 8 and 15 of a 21-day cycle with vinorelbine 30 mg/m^2^ on days 1, 8. 

An objective tumor response was observed in 15 % of the patients; the toxicity was mild to moderate with no grade 3-4 nephrotoxicity or neuropathy. All the patients were HER 2 negative.

Starting from this experience and knowing the worse prognosis of “triple-negative” tumors, our attention was oriented to the possible use of Lipoplatin in this breast disease subset.

It is well known that this kind of breast tumor is associated (both in preclinical and clinical studies) with a high response to traditional chemotherapeutic agents, expecially alkylating agents or Cisplatin.

A further key feature of triple-negative tumors seems to be an angiogenetical activity. It has been inferred that Lipoplatin is endowed with the proprieties of Cisplatin plus the ability of its nanoparticals to target and kill tumor vascular endothelial cells, suggesting that this drugs combines chemotherapeutic and antiangiogenetic properties.

For these reasons, our group is going to start a phase II study in patients with metastatic triple-negative breast disease. The trial is soon to start.

## 8. Conclusions

Lipoplatin represents a new very interesting drug, and its use in advanced lung cancer (NSCLC ) has reached a moderate validation.

Although in some histology subtypes, like adenocarcinoma and undifferentiated NSCLC, the drug's efficacy needs further investigations. 

In breast carcinoma, Lipoplatin represents an interesting drug expecially in HER 2-negative and in triple-negative patients, but its efficacy needs further evidence to be confirmed.

For its characteristics, Lipoplatin could be a possible drug of interest in other cancers like head and neck, gastric, and pancreatic cancer.

## Figures and Tables

**Table 1:** phase I and II studies with lipoplatin.

Author	Chemotherapeutic schema	Study phase	Diseases	Pts N°	Line	OR%	SD%	mTTP	mOS
Stathopoulos et al. [2]	Lipoplatin 25–125 mg/every 2 weeks	Phase I	Various	27	2nd-3rd	11.1%	51.9%		

Froudarakis et al. [3]	Lipoplatin 100–130 mg/m^2^ days 1 and 8, Gemcitabine 1000 mg/m^2^ days 1 and 8 q 21 days	Phase I	NSCLC	13	2nd -3rd	7.6%	15.4%	12 w	29 w

Stathopoulos et al. [4]	Gemcitabine 1000 mg/m^2^ and Lipoplatin 25–125 mg/m^2^ administered as an 8 h i.v. infusion on days 1 and 15, and cycles were repeated every 4 weeks (28 days)	Phase I-II	Pancreatic cancer	24	2nd	8.3%	58.3%		4 m

Ravaioli et al.[5]	Lipoplatin at 100 mg/m^2^ on days 1, 14 in combination with gemcitabine 1000 mg/m^2^ on days 1, 8 in a 28-day cycle	Phase I-II	Various	38	2nd -3rd	23%	65.3%		

Anevlavis et al. [6]	Lipoplatin 100 mg/m^2^ on day 1 in a 14-day cycle	Phase II	NSCLC	19	2nd	5.2%	15.9%		

Mylonakis et al. [7]	Lipoplatin 120 mg/m^2^ days 1 and 8 plus gemcitabine 1000 mg/m^2^ days 1 and 8 every 3 weeks	Phase II	NSCLC	27	2nd cisplatin refractory	22.2%	18.5%		

Farhat et al. [8]	Lipoplatin120 mg/m^2^ (days 1, 8 and 15) and gemcitabine 1000 mg/m^2^ (days 1 and 8) q21-day	Randomized Phase II	NSCLC	47	1st	31.7%	39%		

	Lipoplatin 120 mg/m^2^ (days 1, 8 and 15) and gemcitabine 1000 mg/m^2^ (days 1 and 8) q21-days	41	25.6%	30.8%		

Koukourakis et al. [9]	Vinorelbine 30 mg/m^2^ on (days 1 and 8) while Lipoplatin was administered at the dose of 120 mg/m^2^ on days 1, 8 and 15 q 21d	Phase II	MBC	35		53%	36.7%		

Jehn et al. [10]	Lipoplatin 120 mg/m^2^, 5-FU at 400 mg/m^2^ (day 1), radiotherapy 3.5 Gy fractions on days 2, 3, and 4 in a 7-day schedule	Phase II	Gastric cancer	6 (4 cycles)	1st-line LAD	83%			
				6 (5 cycles)		80%			

**Table 2:** phase III studies with lipoplatin.

Author	Chemotherapeutic schema	Study phase	Diseases	Pts N°	Line	OR%	SD%	mTTP	mOS
Komas et al. [11] and Stathopoubs et al. [12]	Lipoplatin 120 mg/m^2^ on days 1, 8, and 15 plus gemcitabine 1 g/m^2^ on days 1 and 8 in a 21-day cycle	Phase III	NSCLC	33	1st line	25%	23%		

	cisplatin 100 mg/m^2^ on day 1 plus gemcitabine 1 g/m^2^ on days 1 and 8 in a 21-day cycle			26		25%	12%		
Boulikas et al. [13]	100 mg/m^2^/d Lipoplatin as a 4 h i.v. infusion (days 1, 8, 15) plus 1000 mg/m^2^/d 5-FU (days 1–5 continuous infusion) every 21 days	Phase III	SCCHN	25		19%	64%		

	100 mg/ (m^2^ day) cisplatin with prehydration and posthydration (day 1) plus / 1000 mg/m^2^/d / 5-FU (days 1–5 continuous infusion) every 21 days	21		38.8%	50%		

Stathopoulos et al. [14]	Lipoplatin 200 mg/m^2^ plus 135 mg/m^2^ paclitaxel administered on day 1 repeated every 2 weeks	Phase III		114 (79)	1st line	59.70% (59.9%)	36.8%	6.5 (10) m	9 m
	Cisplatin 75 mg/m^2^ 135 mg/m^2^ paclitaxel, administered every 2 weeks		NSCLC (adenocarcinoma and undifferentiated NSCLC)	115 (73)		47.00% (42.5%)	43.5%	6 (8) m	10 m
